# Where’s the Advantage? Mutual Exclusivity Promotes Children’s Initial Mapping, but Not Long-Term Memory, for Words Compared to Other Strategies

**DOI:** 10.3389/fpsyg.2021.686554

**Published:** 2021-09-09

**Authors:** Catherine A. Bredemann, Haley A. Vlach

**Affiliations:** Learning, Cognition, and Development Lab, Department of Educational Psychology, Wisconsin Center for Education Research, University of Wisconsin–Madison, Madison, WI, United States

**Keywords:** word learning, mutual exclusivity, language acquisition, long-term memory, cognitive development

## Abstract

Children frequently apply a novel label to a novel object, a behavior known as the mutual exclusivity bias (MEB). This study examined how MEB affects children’s retention for word mappings. In Experiment 1, preschoolers (*N* = 39; *M_*age*_* = 46.62 months) and adults (*N* = 24; *M_*age*_* = 21.63 years) completed an immediate word mapping task and a delayed retention test. Both samples used MEB during referent selection, but neither group displayed higher retention for words mapped via MEB than words mapped via other referent selection strategies at test. Experiment 2 replicated Experiment 1 with preschoolers (*N* = 85; *M_*age*_* = 47.78 months) and provided evidence against the possibility that interference from multiple words contributed to children’s faster forgetting of word mappings when using MEB. Experiment 3 presented children (*N* = 30; *M_*age*_* = 51.13 months) with an abbreviated version of the task, providing evidence against the alternative hypothesis that cognitive load during learning caused the forgetting observed in Experiments 1 and 2. Taken together, these experiments suggest that MEB supports initial word mapping but may not provide an advantage for long-term retention.

## Introduction

Developmental scientists have long been interested in identifying the mechanisms underlying children’s ability to learn words in situations of referential ambiguity. This interest can be attributed to the significant difficulty involved in determining the referent of a word: theoretically, at any moment in time there is an infinite number of possible referents for a novel word in children’s environment. Despite the difficulty of this task, children readily apply novel words to referents, a behavior known as fast mapping (e.g., Carey and Bartlett, 1978; Mervis and Bertrand, 1994; Mervis et al., 1994; Evey and Merriman, 1998; Horst and Samuelson, 2008; Gurteen et al., 2011; Spiegel and Halberda, 2011; Vlach and Sandhofer, 2012). Thus, many researchers have focused their investigations of children’s word learning on identifying the mechanisms that contribute to fast mapping.

From this work, a variety of theoretical perspectives on the development of children’s fast mapping have emerged. One proposal is that children use strategic constraints or principles to help them reduce the size of the problem space in situations of referential ambiguity (e.g., Markman, 1989; Golinkoff et al., 1994; Mervis and Bertrand, 1994; Woodward, 2000; Mather and Plunkett, 2009). By this account, children’s word mapping is made easy because children possess and utilize the appropriate rules to narrow the number of potential word-referent mappings. One of the most extensively studied constraints is known as the mutual exclusivity bias (MEB). MEB describes children’s and adults’ tendency to assume that an object can have only one linguistic label (e.g., Markman and Wachtel, 1988; Markman, 1989; Merriman et al., 1989; Mather and Plunkett, 2009). When presented with a novel word and two objects, a known object and a novel object, learners will reliably map the novel word to the novel object because the known object is already associated with a specific linguistic label.

Research on children’s use of MEB during word learning has traditionally consisted of variations on a single paradigmatic sequence. First, children are presented with a target novel object along with one or more familiar objects (e.g., a ball). Next, the experimenter presents children with a novel word, asking them to identify the referent for the novel word from among the objects in their visual field (e.g., “Which one is the wug?”). In this task, children reliably select the novel object, indicating to researchers that children use MEB to map the novel word to the novel object. Research using this paradigm has revealed that MEB is employed in the learning of nouns (e.g., Markman, 1989), verbs (e.g., Merriman et al., 1993), and adjectives (e.g., Markman and Wachtel, 1988); within multiple levels of categorical hierarchy (e.g., Markman, 1989; Au and Glusman, 1990); and across development, in infancy (e.g., Halberda, 2003; Mather and Plunkett, 2009), early childhood (e.g., Markman, 1990; Merriman et al., 1993) and adulthood (e.g., Markman and Wachtel, 1988; Au and Glusman, 1990).

Although there have been many studies conducted on children’s use of MEB, there is a striking limitation of the MEB literature: assessments of word mapping are most often administered at an immediate test, directly following the learning event (e.g., Merriman et al., 1989; Au and Glusman, 1990; but see Horst and Samuelson, 2008; Axelsson et al., 2018). That is, these studies examine children’s mapping, but not retention, of new words learned via MEB. In addition, because children’s tendency to use MEB to map words to objects is so robustly demonstrated in the literature, it has been assumed that it is inherently supportive of children’s word learning. However, it remains largely unclear whether and how MEB supports word learning across time. This gap in our understanding of MEB prompted our question: Does the use of MEB, in addition to facilitating word mapping, also promote the long-term retention of words? And, in particular, does it actually benefit children’s retention to a greater degree than other word mapping strategies?

One hypothesis is that MEB promotes the retention and retrieval of word mappings to a greater degree than other strategies of resolving referential ambiguity, such as associative learning (e.g., McMurray et al., 2012). For instance, MEB may promote the long-term retention of newly learned words because MEB supports the initial encoding of word mappings. MEB constrains the number of possibilities of a referent for a new word; thus, it could reduce the amount of information that learners must attend to and encode during the referent selection process. That is, by allowing learners to focus on one association between a word and referent, MEB may allow for a deeper encoding of that word mapping. Indeed, in theories of human memory, encoding strength is one of the guiding factors determining whether learners can retain and retrieve knowledge across time (e.g., Soderstrom and Bjork, 2015).

By another hypothesis, MEB may not support long-term retention of words to a greater degree than other referent-selection strategies. Other referent selection strategies lead to forgetting after learning, such as associative learning and ostensive naming (e.g., McMurray et al., 2012; Vlach and Sandhofer, 2012), and thus words mapped via MEB may follow a similar pattern of forgetting. Moreover, MEB may be an in-the-moment phenomenon, useful for mapping words to objects yet entirely impartial to the formation of long-term memory for new words. For instance, learners often engage in less cognitive effort during learning when word mapping is easy (Vlach and Sandhofer, 2014). Although immediate performance may be strong because of the ease of the task, long-term retrieval of the information often suffers due to diminished cognitive effort in encoding occurring during learning. Indeed, immediate performance in a word learning task does not always equate to performance over time (Vlach et al., 2012; Vlach and Sandhofer, 2014). Thus, although MEB reduces the difficulty of the task of referent selection, the relative cognitive ease of using this bias may result in weaker retrieval of word mappings in the future.

An alternative theoretical account of MEB suggests that this behavior is guided by endogenous, novelty-seeking preferences, rather than the application of rules (Horst et al., 2011a; Mather and Plunkett, 2012; Dysart et al., 2016). If MEB is indeed a result of novelty-seeking, this might lead to weak encoding of word mappings. Research has shown that novelty-driven learning does not necessarily result in strong long-term performance (Kucker, 2013). For instance, one study in this line of work (Horst and Samuelson, 2008) examined 24-month-old infants’ ability to retain words mapped via MEB after a 5-min delay. The results showed that infants had no retention of words mapped via MEB without the addition of memory supports (e.g., enhancing the salience of novel words), and the authors suggested that the novelty-seeking behavior of MEB did not produce a strong enough representation to retain words across a delay.

Although a small number of experiments have looked at how MEB affects young children’s word learning after a brief delay (e.g., Horst and Samuelson, 2008; Bion et al., 2013), there are limitations in using these data to answer our particular research question. First, infants in these studies often show little or no retention regardless of how they map labels (e.g., via MEB, ostensive naming, etc.) without receiving additional memory supports. It could be that infants at 24 months do not have strong enough long-term memory abilities to retain a word mapped in one moment of referential ambiguity and with no additional information, feedback, or support. Second, these studies have not directly compared children’s use of MEB with their use of another mapping strategy in moments of referential ambiguity. Indeed, these studies use ostensive naming as a comparison condition, but ostensive naming typically does not require resolving referential ambiguity.

### Current Study

In the current work, we tested the above hypotheses by investigating whether MEB provides an advantage for long-term retention of word mappings. That is, we examined children’s retention and forgetting of labels mapped via MEB relative to their retention and forgetting of labels that were not mapped using MEB in order to ascertain whether MEB benefits children’s word learning above and beyond other word mapping strategies.

To isolate how MEB uniquely affects memory processes during referent selection, a paradigm which induced learners’ use of either MEB or another referent-selection strategy was needed. This would provide a fairer comparison than pitting MEB against an ostensive naming condition, as ostensive naming condition would not require resolving ambiguity. Thus, the word-mapping portion of the current study employed the classic MEB research paradigm, but with a critical alteration: we provided learners with two opportunities to map novel labels to a specific novel object. We expected learners to use MEB to map a novel word to a novel object at its first occurrence. At the second occurrence, we expected that learners would be less likely to use MEB to map a novel label to that object, as the object already has a label from the first trial. Instead, we expected children to use another referent-selection strategy, such as applying a novel label to a novel, but familiar object (e.g., applying “wug” to a novel picture of a cat), responding randomly, using phonological overlap between the novel word and known words, visual preference of the images, etc. That is, because the novel object and known object have labels on the second mapping trial, MEB no longer provides a strategy for word mapping and thus children must apply other strategies. The strength of this protocol is that it resulted in a number of learning trials in which learners used MEB as well as a number of trials in which they likely used another referent selection strategy. The limitation of this protocol is that we do not know which of the many strategies children could use when both objects have a label. However, in this way, we could directly compare how MEB affects retention across a delay compared to all other possible mapping strategies, and ascertain whether MEB provides an advantage for word learning.

In addition, we consider development to be an important factor in MEB use and retention of novel words, as it could be that older learners with greater memory skills employ MEB in different manners than younger learners (for a discussion, see O’Connor and Riggs, 2019). Studies of referent selection and long-term memory have typically included only very small age ranges during infancy (e.g., Horst and Samuelson, 2008; Bion et al., 2013). To address these limitations and answer our research questions, we examined older children’s (i.e., preschoolers) and adults’ retention of words learned via MEB.

In Experiment 1, we presented preschool-aged children and adults with a typical MEB word-learning task, which included a 5-min retention period between the learning and testing phases. A 5-min delay between learning and testing required participants to access word-object pairings from long-term memory, affording an analysis of how words mapped via MEB vs. words likely not mapped via MEB are retained. In Experiment 2, we replicated the results of Experiment 1 in children and examined whether interference was contributing to the results obtained in Experiment 1. In Experiment 3, we presented children with an abbreviated version of the task to examine whether general cognitive load during learning was contributing to the results of Experiments 1 and 2.

## Experiment 1

### Methods

#### Participants

Thirty-nine preschool-aged children (*M*age = 46.62 months, *SD* = 9.99, Range: 25–69 months; 21 girls) and 24 adults (*M*age = 21.63 years, *SD* = 6.34, Range: 18–45 years; 22 females) participated in the experiment. Effect sizes were gathered from previous studies on referent selection via MEB with children at 2 years of age, the youngest in this sample, which had consistently large effect sizes (*d*s > 1.0; Horst and Samuelson, 2008; Casler, 2014). To be conservative in determining a sample size, we used a smaller effect size in the large effect category: *d* = 0.90. A power analysis for a two-tailed *t*-test, with α = 0.05, revealed that we would need at least 21 participants to have 80% power to observe an effect. Thus, we decided to collect data for 4 months or until we reached 21 participants (if we did not reach this number within the 4-month period).

Children at this point in development were chosen because they are actively learning new words and have been studied in prior studies of MEB (e.g., Markman, 1989; Merriman et al., 1989), although not at a delayed test. Infants often show little or no retention regardless of how they map labels (e.g., via MEB, ostensive naming, etc., Horst and Samuelson, 2008), and thus we targeted children older than 24 months. Children were recruited through a preschool database belonging to a child development laboratory at University of Wisconsin–Madison. Children were from primarily middle- to upper-SES families. An additional 24 children participated but were not included in the final sample due to fussiness, inability to follow instructions to complete the experiment, or experimenter error. After completing the study, children received a storybook for their participation. Adult participants were recruited from a subject pool of undergraduate students enrolled in introductory psychology courses at University of Wisconsin–Madison. After completing the study, adult participants received course credit for their participation. Informed consent was obtained from all participants.

#### Stimuli and Apparatus

**FIGURE 1:**
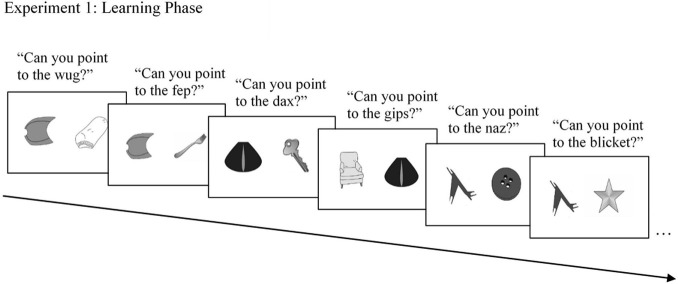
Examples of stimuli used in the learning phase of Experiment 1. In Experiment 1, participants were presented with two novel labels in two consecutive learning trials for the same target novel object, along with familiar objects as foils. This allowed participants a first opportunity to use MEB, and immediately following, another situation of referential ambiguity when children were unlikely to use MEB.

Participants were presented with 40 familiar objects, 40 novel objects, and 40 novel linguistic labels during the experiment (for examples, see Figure 1). The objects were presented on an iPad or laptop using an electronic presentation application. The novel linguistic labels and testing prompts were provided by the experimenter.

Familiar objects were line drawings of objects that would be known to the children (e.g., key and chair). These objects were cross-referenced with the MacArthur Communicative Development Inventory of children’s productive vocabulary (MCDI-III; Fenson et al., 1994) to ensure that even the youngest children in the study would know and be able to produce the linguistic labels for these objects. The novel objects were line drawings generated in graphic-editing software. To minimize differences in saliency across objects in order to prevent saliency from affecting children’s responses, the novel objects were piloted to a group of adult participants (*N* = 24) who rated each object pair for similarity and complexity. These participants did not participate in Experiment 1. Objects rated as significantly similar or complex relative to other objects were not used in the study. The novel linguistic labels were one- and two-syllable pseudo-words that followed the phonotactic probabilities of English (e.g., “wug” and “dax”).

#### Design and Procedure

The experiment consisted of three phases: a learning phase, a retention phase, and a testing phase. In the learning phase, participants were introduced to two novel labels for each novel object. In the testing phase, participants’ memory for an object was tested using one of the two labels presented during the learning phase.

##### Learning phase: referent selection learning trials

There were two learning trials for each of the 20 target novel objects, for a total of 40 learning trials in the learning phase. Each learning trial presented children with two objects: a familiar object and a target novel object (Figure 1). Participants were presented with a first learning trial (referred to as the “M1” trial) containing a familiar object (e.g., a key) and a novel object. Participants were asked by the experimenter to select the referent for a novel word (e.g., “Which one is the wug?”). Immediately following the first learning trial was a second learning trial (referred to as the “M2” trial), again with one pair of objects, this time comprised of a new familiar object (e.g., a sock) paired with the same target novel object as was shown in the previous trial. Participants were again asked to select the referent for a novel word, one that differed from the word previously presented with the specific novel object (e.g., “Which one is the dax?”). Thus, each target novel object was presented with two distinct novel words. The orientation of the familiar and novel objects on the screen was counterbalanced across learning trials (e.g., the target novel object was not always on the left side of the screen). Transitions between trials were controlled manually by the experimenter, so that participants could take their time to respond to the prompt. Participants received no feedback on either the first or second learning trials as to whether their responses were correct. Participants indicated their selection by pointing at an object on the screen, and the experimenter recorded participants’ answers on paper.

##### Retention phase: 5-min delay

This phase consisted of a 5-min delay period. During this delay, children participated in a short, unrelated activity or game (e.g., putting stickers on paper), and adults were able to pass time freely on an iPad or another mobile device.

##### Testing phase: word-referent mapping memory task

**FIGURE 2:**
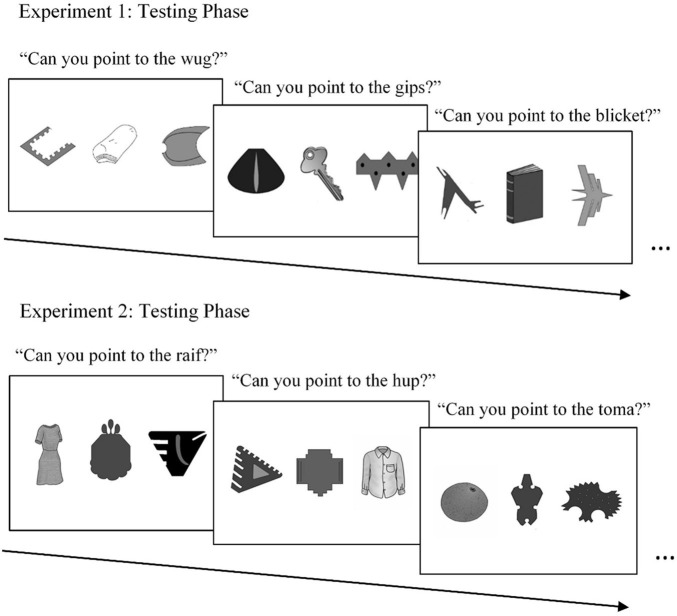
Examples of stimuli used in the testing phases of Experiments 1 and 2. In both experiments, the testing phase followed the 5-min retention phase. On each test trial, participants were asked to select the referent for one of the two novel words presented in the learning phase assigned to each target novel object.

Each of the 20 trials of the testing phase presented participants with three objects (Figure 2). One object on the screen was a target novel object that was presented in two consecutive trials in the learning phase. The second object on the screen was a familiar object which was presented in the learning phase alongside the target object. The familiar object shown at test was shown on either the first or second learning trial in which the specific target novel object was presented (e.g., a key). Whether the familiar object was one presented from the first learning trial (M1) or the second learning trial (M2) for an object was counterbalanced across the 20 test trials. The third object was a novel object that had not been presented in any other part of the experiment, to control for familiarity. The orientation of the three types of objects was also counterbalanced across the 20 test trials (e.g., the target novel object was not always on the far left side of the screen). Each novel object was tested once, in the same order presented during the learning phase to ensure equivalent retention intervals for each of the objects.

On each test trial, children were asked to select the referent for one of the two novel words presented in the learning phase assigned to the novel target object (e.g., “Which one is the dax?”). In 10 of the 20 test trials, children were asked to select the referent for the first word presented for the target object in the learning phase (M1), and in the other 10 test trials, children were asked to select the referent for the second word (M2). That is, children were tested on only one of the two mappings made for each object presented in the learning phase. Children indicated their selection by pointing to an object on the screen, and the experimenter recorded children’s selections on paper.

### Results and Discussion

In the following analyses, we examined whether participants: (a) used MEB during the learning phase; and (b) had differential retention of words mapped via MEB vs. words mapped via a lesser degree of MEB or without using MEB (i.e., first vs. second word mappings in the learning phase). Because past experiments have demonstrated that MEB supports learners’ referent selection at an immediate test, we hypothesized that MEB would facilitate the process of accessing word mappings from long-term memory.

#### Performance in the Learning Phase

**FIGURE 3:**
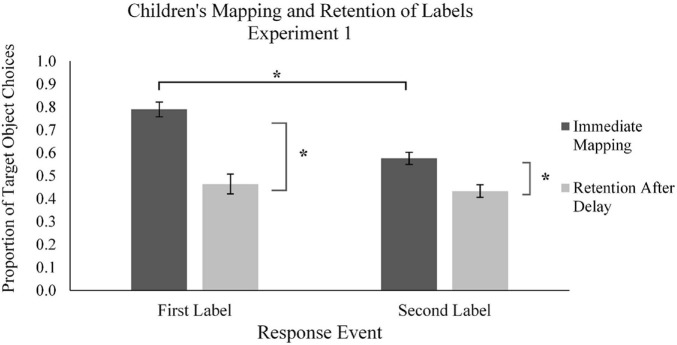
Mean proportion of children’s target novel object choices in the learning phase (immediate mapping) and testing phase (retention after delay) for first and second labels in Experiment 1. Error bars represent one standard error, * indicates significant difference in performance, *p* < 0.05.

**FIGURE 4:**
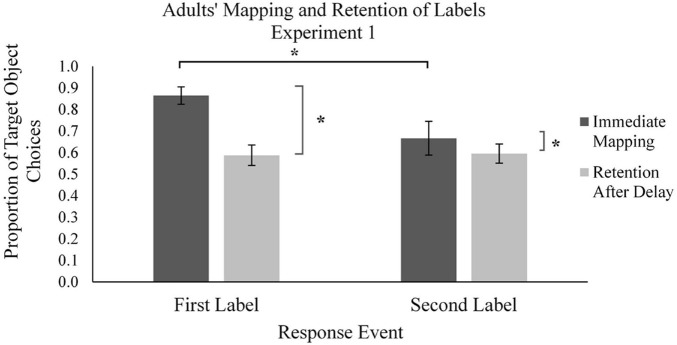
Mean proportion of adults’ target novel object choices in the learning phase (immediate mapping) and testing phase (retention after delay) for first and second labels in Experiment 1. Error bars represent one standard error, * indicates significant difference in performance, *p* < 0.05.

Participants’ performance during the learning phase was measured as the proportion of target novel objects selected across the 40 learning trials (chance performance was 0.50, as novel objects were presented with one familiar object each). We defined learners’ use of MEB during the learning phase as the mapping of novel words to novel objects rather than to familiar objects. In analyzing participants’ performance in the learning phase, the use of MEB in applying novel labels to objects was compared across the two types of trials: the first mapping (M1) and second mapping (M2) trials for each novel object (Figures 3, 4).

We first conducted a mixed-design, repeated-measures ANOVA with age group (children and adults) as a between-subjects factor and mapping trial type (M1 vs. M2) as a within-subjects factor. This test revealed no effect of age group and no interaction effects, but we found a main effect of mapping, *F*(1,61) = 39.73, *p* < 0.001, η*_*p*_^2^* = 0.394. In anticipation of an age effect at the post-test, we conducted separate analyses of children’s and adults’ mapping performance in the learning phase.

We started by examining children’s word mapping behavior using a series of two-tailed *t*-tests (Figure 3). Children mapped the first labels to target novel objects on a majority of the M1 trials (*M_*proportion of novel object choices*_* = 0.79, *SD* = 0.20). Children’s performance was significantly different from chance, *t*(38) = 9.01, *p* < 0.001, *d* = 1.44, suggesting that children were using MEB to map words to objects in M1 trials. In contrast, children’s mapping of the second label to the target object in M2 trials (*M_*proportion of novel object choices*_* = 0.58, *SD* = 0.27) was not significantly different from chance, *p* > 0.05. Critically, there was a significant difference in children’s mapping between M1 and M2 labels, *t*(38) = 5.76, *p* < 0.001, *d* = 0.923, suggesting that children were relying on MEB when mapping the first novel words to objects, but not the second novel words.

We conducted the same analyses of adults’ performance during the learning phase (Figure 4). We observed that adults, like children, mapped the majority of novel words to target novel objects during M1 trials (*M_*proportion of novel object choices*_* = 0.86, *SD* = 0.20). Adults also mapped the majority of novel words to the target novel object during the M2 trials (*M_*proportion of novel object choices*_* = 0.67, *SD* = 0.39). Adults’ performance was significantly above chance of 0.5 in M1 trials, *t*(23) = 9.04, *p* < 0.001, *d* = 1.85, and in M2 trials, *t*(23) = 2.12, *p* = 0.045, *d* = 0.432, suggesting that adults used MEB to disambiguate both labels. However, there was a significant difference between the first and second mapping, *t*(23) = 3.44, *p* = 0.002, *d* = 0.702, suggesting that adults used MEB to a lesser degree on the second mapping.

#### Performance in the Testing Phase

Participants’ performance during the testing phase was measured as the proportion of target novel objects selected across all 20 test trials (chance performance was 0.33, as target novel objects were presented with one familiar object and one distractor novel object each). We first calculated participants’ retention scores on test trials of each type (M1 vs. M2 trials). We then conducted a one-way ANOVA with age group (children and adults) as a between-subjects factor and total test performance as the dependent variable. This test revealed a main effect of age group, *F*(1,61) = 10.14, *p* = 0.002, η*_*p*_^2^* = 0.143. We further analyzed performance of children and adults for retention of novel labels.

Contrary to our first hypothesis, at the 5-min delayed test children did not have greater memory performance for the words mapped via MEB relative to other strategies (Figure 3). That is, retention of M1 labels [*M_*proportion remembered*_* = 0.46, *SD* = 0.16; comparison to chance at 0.33, *t*(38) = 4.97, *p* < 0.001, *d* = 0.797] and retention of M2 labels [*M_*proportion remembered*_* = 0.43, *SD* = 0.17; comparison to chance at 0.33, *t*(38) = 3.64, *p* = 0.001, *d* = 0.582] did not differ significantly, *p* > 0.10. Use of MEB, therefore, did not provide an advantage for word learning after a delay of just 5 min.

Indeed, a further examination of the forgetting (i.e., difference scores) of labels across the two time points revealed that MEB use did not provide an advantage for later retention, but it also may have induced more rapid forgetting. First, we observed significant differences across time between children’s mapping and retention for both M1 labels [*t*(38) = 12.01, *p* < 0.001, *d* = 1.923] and for M2 labels [*t*(23) = 4.30, *p* < 0.001, *d* = 0.689]. This indicates that, although children’s performance at test was above chance, forgetting occurred in both conditions, as was expected. However, a within-subjects *t*-test on the difference scores across the two time points for the M1 labels (*M_*proportion forgotten*_* = 0.33, *SD* = 0.17) and M2 labels (*M_*proportion forgotten*_* = 0.14, *SD* = 0.21) revealed a significant difference, *t*(38) = 4.71, *p* < 0.001, *d* = 0.755. We also examined children’s forgetting (i.e., difference scores) of M1 labels for trials in which children chose the novel object during the learning phase. We compared the difference score for these trials (*M*_*diffscore*_ = 3.95, *SD* = 1.88) to 0 (no forgetting) using a one-sample *t*-test; this revealed a significant difference, *t*(38) = 13.14, *p* < 0.001, *d* = 2.103. Taken together, these results suggest that children more rapidly forgot the words which they had mapped using MEB.

Adults’ performance during the testing phase was analyzed in the same manner as the children’s performance (Figure 4). Contrary to our first hypothesis, at the 5-min delayed test adults did not have greater memory performance for labels that were more frequently mapped using MEB than labels that were more infrequently mapped using MEB. That is, retention of M1 labels [*M_*proportion remembered*_* = 0.59, *SD* = 0.23; comparison to chance at 0.33, *t*(23) = 5.35, *p* < 0.001, *d* = 1.09] and retention of M2 labels [*M_*proportion remembered*_* = 0.60, *SD* = 0.22; comparison to chance at 0.33, *t*(23) = 5.92, *p* < 0.001, *d* = 1.21] did not differ significantly, *t*(23) = −0.23, *p* > 0.10. We also examined adults’ forgetting of labels across the two time points. A within-subjects *t*-test on the difference scores across the two time points for the M1 labels (*M_*proportion forgotten*_* = 0.28, *SD* = 0.22) and M2 labels (*M_*proportion forgotten*_* = 0.07, *SD* = 0.32) revealed a significant difference, *t*(23) = 3.70, *p* = 0.001, *d* = 0.755. This finding suggests that adults more rapidly forgot the words which they had mapped using MEB. Taken together, these results suggest that MEB supports initial mappings of words to objects in situations of referential ambiguity, but this linguistic constraint may not support long-term language learning. That is, from the preschool years to adulthood, using MEB in the moment of learning did not result in stronger retention of word-object associations across time.

There are two possible explanations for these findings. First, it may be that the use of MEB is a weak form of encoding. That is, it could be that MEB behavior is driven by a novelty-seeking preference that induces shallow encoding, as suggested by alternative accounts of MEB (e.g., Horst et al., 2011b; Mather and Plunkett, 2012; Dysart et al., 2016). Learners presented with a novel word will quickly assume that the word is likely to refer to a novel object; upon finding the target novel object, however, the novelty-seeking has been accomplished and learners disengage from the task, failing to deeply encode the association between the specific novel word and its referent.

Alternatively, it could be that the results we observed were driven by the effects of interference, due to the presentation of two labels for the same object in immediate succession. The protocol in the current study was structured to induce the differential use of MEB across multiple novel labels for a single object. However, the presentation of two novel labels in immediate succession may have confounded the results that we obtained. In other words, participants’ lower retention for first labels could be due to weaker encoding of the first label caused by interference from introduction of a second label so soon after the first. It is therefore difficult to ascertain whether participants’ significant forgetting of words mapped via MEB was a direct consequence of MEB use or whether this pattern of results was a consequence of the experimental design.

We examined the potential effect of interference in Experiment 2. We presented children with varying degrees of interference between the moment of word mapping and the delayed post-test. There were three experimental conditions: an Immediate Interference condition, which served as a replication of Experiment 1; a Delayed Interference condition, in which the introduction of a second label was delayed in time across the learning phase; and a No Interference condition, in which children were only presented with one label for each novel object. We predicted that, across the three conditions, there would be no difference in children’s retention and forgetting of labels mapped via MEB. Based on the results of Experiment 1, which demonstrated a large effect of differential forgetting between M1 and M2 labels, it appears children’s memory for labels mapped via MEB may be much more susceptible to decay. Because children across all conditions of Experiment 2 were presented with the first label for each object in the same way, we expected children to use MEB to map the first label to the novel object across all conditions in a similar manner as children in Experiment 1.

If results of Experiment 2 demonstrated that performance in the Delayed Interference and No Interference conditions was not significantly better than in the Immediate Interference condition, we could conclude that the difference in children’s forgetting seen across the M1 and M2 labels in Experiment 1 was due to the failure of MEB to support children’s learning of words, and not due to the interference of multiple labels.

## Experiment 2

### Methods

#### Participants

Eighty-five preschool-aged children (*M*_*age*_ = 47.78 months, *SD* = 9.23, Range: 27–75 months; 43 girls) participated in the experiment. Effect sizes were gathered from previous studies on referent selection via MEB with children at 2 years of age, the youngest in this sample, which had consistently large effect sizes f (*d*s > 1.0; Horst and Samuelson, 2008; Casler, 2014). To be conservative in determining a sample size, we used a smaller effect size in the large effect category: *d* = 0.90. A power analysis for a two-tailed *t*-test, with α = 0.05, revealed that we would need at least 21 participants in each of the three conditions to have 80% power to observe an effect. Thus, we decided to collect data for 4 months or until we reached 21 participants in each between-subjects condition (if we did not reach this number within the 4-month period). These children had not participated in Experiment 1. Children were recruited through a lab preschool database at University of Wisconsin–Madison. An additional 45 children participated but were not included in the final sample due to fussiness, inability to follow instructions to complete the experiment, or experimenter error (this is consistent with other studies of mutual exclusivity with very young children, e.g., Bion et al., 2013). All children received a storybook as a thank-you for their participation. Informed consent was obtained from all participants.

#### Stimuli and Apparatus

The same familiar and novel objects from Experiment 1 were presented in the Immediate Interference and the Delayed Interference conditions, and the No Interference condition included these as well as 20 more familiar and novel objects each. In all three conditions, the same novel linguistic labels from Experiment 1 were used. The experiment was presented on an iPad or laptop using a presentation application. Children’s selections were recorded by the experimenter on paper.

#### Design and Procedure

Children were presented with a word-mapping task consisting of a learning phase, a retention phase, and a testing phase. Children were randomly assigned to one of three between-subjects conditions, which varied in the presentation of the second label: an Immediate Interference condition; a Delayed Interference condition; and a No Interference condition. Random assignment resulted in the following samples for each condition: Immediate Interference (*N* = 27; *M_*age*_* = 46.67 months; 16 girls); Delayed Interference (*N* = 29; *M_*age*_* = 49.48 months; 12 girls); and No Interference (*N* = 29; *M_*age*_* = 47.10 months; 15 girls).

##### Learning phase: referent selection learning trials

**FIGURE 5:**
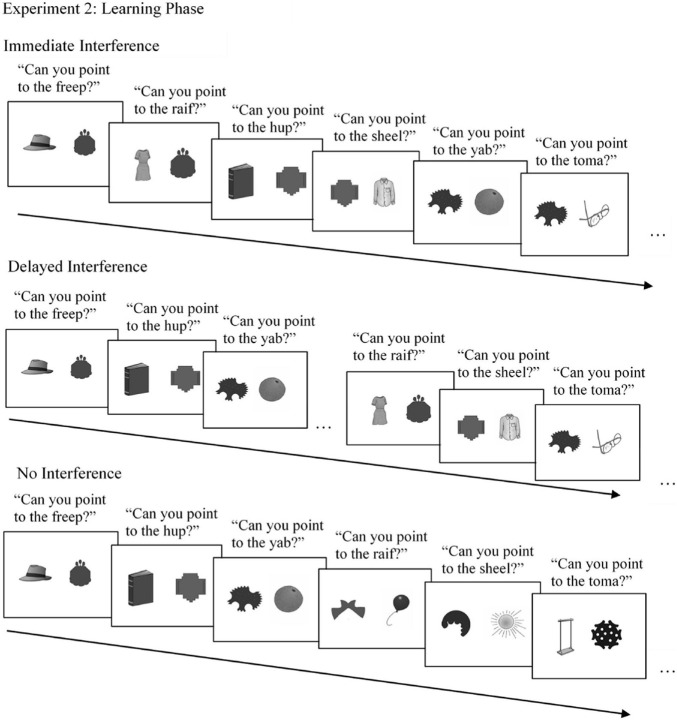
Examples of stimuli used in the learning phases of Experiment 2. In the three conditions of Experiment 2, protocol of Experiment 1 was replicated (Immediate Interference), the second mapping event for each object occurred after a delay (Delayed Interference), or no second mapping event occurred (No Interference), to examine the potential role of interference in children’s retention of word mappings.

In the Immediate Interference condition, the same word-learning paradigm from Experiment 1 was used to examine whether the results of the first experiment replicated (Figure 5). The second label for the target object was presented immediately following the first label for the target object.

In the Delayed Interference condition, the degree of interference was manipulated by altering the presentation timing of the second labeling event (Figure 5). The introduction of the second label for each novel object was delayed until the second half of the learning phase. That is, one learning trial for each novel object and its first label was presented in immediate succession, until all target novel objects had been shown (M1 trials). Then, when all objects had been presented once with a first label, the presentation cycled through each object once more, in order, and presented children with a second label for each target novel object (M2 trials). This presentation procedure reduced the likelihood of the second label interfering with children’s encoding of the first label to long-term memory, due to the temporal gap between presentations.

In the No Interference condition, children were presented with a learning phase which consisted of target novel objects that were only labeled once (Figure 5). That is, there were 40 novel objects and 40 novel labels; each word and object was only presented on one learning trial. Thus, children in this learning condition heard the same number of words and saw the same number of trials as the children in Experiment 1 and in the other two conditions of Experiment 2. This presentation procedure eliminated any possible interference from a second label by presenting children with only one label to accompany each novel object.

##### Retention phase: 5-min delay

Same as Experiment 1.

##### Testing phase: word-referent mapping memory task

Same as Experiment 1 (Figure 2).

### Results and Discussion

Experiment 2 was designed to replicate Experiment 1 and determine whether interference after learning led to faster forgetting of words mapped via MEB. As in Experiment 1, we examined whether children: (a) used MEB during the learning phase; and (b) had differential retention of words mapped via MEB vs. words mapped via a lesser degree of MEB or not using MEB (i.e., first vs. second word mappings in the learning phase).

#### Performance in the Learning Phase

Children’s performance during the learning phase across all three conditions was measured as the proportion of target novel objects selected across the 40 learning trials (chance performance was 0.50, as novel objects were presented with one familiar object each). As in Experiment 1, we defined learners’ use of MEB as the mapping of novel words to novel objects rather than to familiar objects during the learning phase. In analyzing participants’ performance in the learning phase, their use of MEB in applying novel labels to objects was compared across the two types of trials: the first mapping (M1) and second mapping (M2) trials.

**FIGURE 6:**
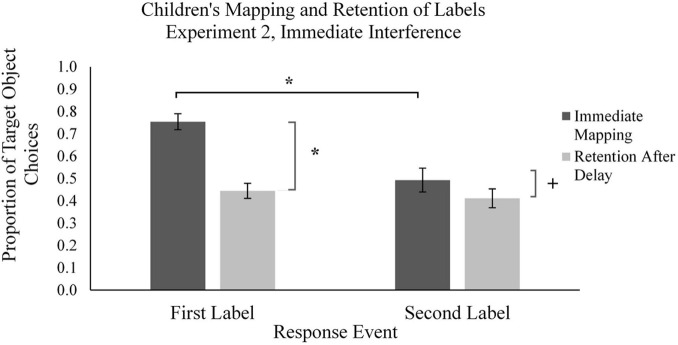
Mean proportion of children’s target novel object choices in the learning phase (immediate mapping) and testing phase (retention after delay) for first and second labels in the Immediate Interference condition in Experiment 2. Error bars represent one standard error, * indicates significant difference in performance, *p* < 0.05, ^+^indicates marginally significant difference, *p* = 0.09.

We started by examining children’s word mapping behavior in the Immediate Interference condition, which served as a replication condition for Experiment 1, using a series of two-tailed *t*-tests (Figure 6). Results revealed that children mapped the first labels to target novel objects on a majority of the M1 trials (*M_*proportion of novel object choices*_* = 0.75, *SD* = 0.19). Children’s performance was significantly different from chance, *t*(26) = 7.09, *p* < 0.001, *d* = 1.36, suggesting that children were using MEB to map words to objects in M1 trials. In contrast, children’s mapping of the second label to the target object in M2 trials (*M_*proportion of novel object choices*_* = 0.49, *SD* = 0.28) did not differ from chance, *p* > 0.10. As in Experiment 1, there was a significant difference in children’s mapping between M1 and M2 labels, *t*(26) = 5.55, *p* < 0.001, *d* = 1.067, suggesting that children were relying on MEB when mapping the first novel words to objects, but not the second novel words. This pattern of findings replicates the behavior observed in Experiment 1.

**FIGURE 7:**
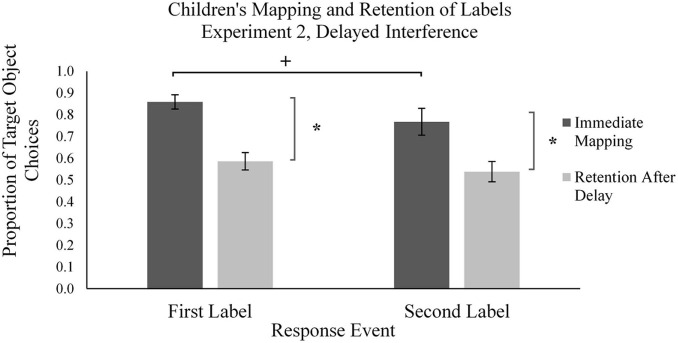
Mean proportion of children’s target novel object choices in the learning phase (immediate mapping) and testing phase (retention after delay) for first and second labels the Delayed Interference condition in Experiment 2. Error bars represent one standard error, * indicates significant difference in performance, *p* < 0.05, ^+^indicates marginally significant difference in performance, *p* = 0.06.

We conducted the same analyses with the children in the Delayed Interference condition (Figure 7). Results revealed that these children also mapped the majority of novel words to the target novel objects during M1 trials (*M_*proportion of novel object choices*_* = 0.85, *SD* = 0.18). Similarly, during M2 trials, children mapped the majority of novel words to the target novel object (*M_*proportion of novel object choices*_* = 0.77, *SD* = 0.33). Children’s performance was significantly above chance of 0.5 in M1 trials, *t*(28) = 10.99, *p* < 0.001, *d* = 2.04, and in M2 trials, *t*(28) = 4.32, *p* < 0.001, *d* = 0.80, suggesting that children used MEB for both labels. However, there was a marginally significant difference between the first and second mapping, *t*(28) = 2.00, *p* = 0.056, *d* = 0.371, suggesting that children used MEB to a lesser degree on the second mapping.

**FIGURE 8:**
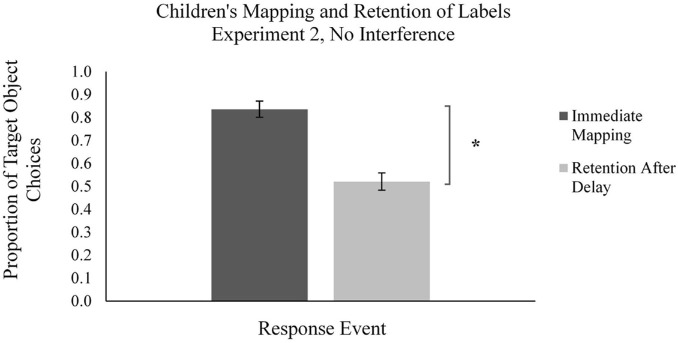
Mean proportion of children’s target novel object choices in the learning phase (immediate mapping) and testing phase (retention after delay) for labels in the No Interference condition in Experiment 2. Error bars represent one standard error, * indicates significant difference in performance, *p* < 0.05.

Finally, we examined how children performed in the learning phase in the No Interference condition (Figure 8). Because no second labels were presented in this condition, we examined children’s mapping of novel labels to novel objects on a one-to-one basis (i.e., all trials were treated as M1 trials). Children in this condition mapped the novel labels to target novel objects on a majority of trials (*M_*proportion of novel object choices*_* = 0.83, *SD* = 0.19). Children’s performance was significantly different from chance, *t*(28) = 9.60, *p* < 0.001, *d* = 1.78, suggesting that children were using MEB to map words to objects in M1 trials.

#### Performance in the Testing Phase

Children’s performance during the testing phase was measured as the proportion of target novel objects selected across all 20 test trials (chance performance was 0.33, as target novel objects were presented with one familiar object and one distractor novel object each). We first calculated children’s retention scores on test trials of each type (M1 vs. M2 trials), and then further analyzed children’s retention performance for each label.

As in Experiment 1, children did not have greater memory performance for the words mapped via MEB after a delayed test. A series of *t*-tests revealed that children in both learning conditions involving multiple labels – Immediate Interference (Figure 6) and Delayed Interference (Figure 7) – did not have better retention for first-label mappings than second-label mappings, *p*s > 0.10. That is, no significant differences were observed between retention rates of the two label types, M1 and M2.

Further examination of the forgetting (i.e., difference scores) of labels across time in each condition revealed differences across conditions in children’s forgetting of M1 and M2 labels. In the Immediate Interference condition, as in Experiment 1, we observed a significant difference across time between children’s mapping and retention for M1 labels [*t*(26) = 8.47, *p* < 0.001, *d* = 1.631]; we observed a marginal difference in performance across time for M2 labels [*t*(26) = 1.97, *p* = 0.059, *d* = 0.379] (Figure 6). This indicates that, although children’s performance at test was above chance, forgetting occurred in both conditions, as was expected. Furthermore, in the Immediate Interference condition we also replicated the results of the forgetting analysis from Experiment 1: a within-subjects *t*-test on the difference scores across the two time points for the M1 labels (*M_*proportion forgotten*_* = 0.31, *SD* = 0.19) and M2 labels (*M_*proportion forgotten*_* = 0.08 *SD* = 0.21) revealed a significant difference, *t*(38) = 4.53, *p* < 0.001, *d* = 0.871. This finding suggests that children more rapidly forgot the words which they had mapped using MEB.

We performed these same forgetting analyses on children’s performance in the Delayed Interference condition and found children’s forgetting across time to be similar to the other two samples of children (Figure 7). That is, we observed a significant difference across time between children’s mapping and retention for M1 labels [*t*(28) = 7.95, *p* < 0.001, *d* = 1.477]; we also observed a significant difference in performance across time for M2 labels [*t*(28) = 3.67, *p* = 0.001, *d* = 0.682]. However, we did not find a significant difference between the difference scores across the two learning conditions, *p* > 0.10. This indicates that, whether children were presented with the second label immediately after the first, or whether they were presented with the second label after a delay, their rates of forgetting across time did not differ.

**FIGURE 9:**
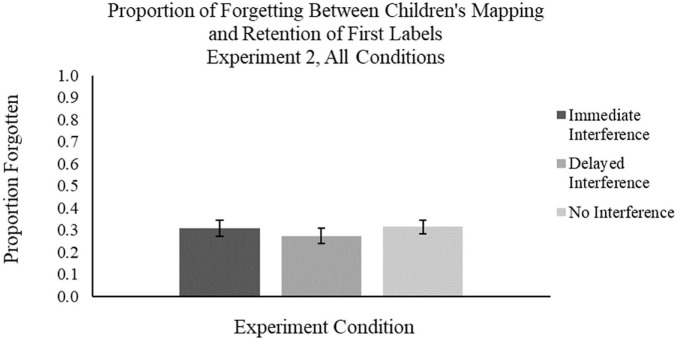
Mean proportion of children’s forgetting of novel labels from learning phase to testing phase, by condition. Error bars represent one standard error.

Finally, we examined differences between the forgetting scores for M1 labels in the Immediate Interference and Delayed Interference conditions relative to the No Interference condition (Figure 9). By including the No Interference condition in Experiment 2, we were able to compare forgetting rates across all conditions, in order to rule out the possibility that the interference from a second label was the mechanism for children’s significant forgetting of labels mapped via MEB. Children in the No Interference condition also exhibited significant forgetting between word mapping and testing, *t*(28) = 9.90, *p* < 0.001, *d* = 1.838 (Figure 8). Importantly, the forgetting rates across all conditions for M1 labels did not differ significantly by independent samples *t*-tests, *p*s > 0.10. That is, children’s forgetting of labels mapped most often via MEB was not due to the immediate or delayed interference of a second label. This pattern of results remains even after we examined only the test trials which corresponded to learning trials in which children had successfully mapped the label to the novel object, *p* > 0.10. Taken together, these results replicate Experiment 1 and provide evidence against interference as the primary cause of forgetting of words mapped via MEB. The implications of these findings will be discussed in greater detail in the section “General Discussion.”

In Experiment 3, we examined another alternative explanation for the results observed in Experiments 1 and 2: cognitive load during learning. The learning phase of Experiments 1 and 2 required children to map 40 novel labels to objects. This large amount of new information may have taxed their cognitive resources (e.g., working memory capacity, inhibition of proactive interference from other newly learned words, decision-making on what non-MEB strategy to use on the M2 trials, etc.) and led to forgetting MEB mapped words between learning and post-test. Thus, in Experiment 3 we reduced the cognitive demands of the learning phase; children were presented with only four MEB trials during learning. If children demonstrated forgetting between learning and the post-test, as was observed in Experiments 1 and 2, this would provide evidence against general cognitive load as an explanation for the findings in this work. If we observed equivalent performance during learning and the post-test, this would suggest that the cognitive demands of the experimental design were causing the forgetting.

## Experiment 3

### Methods

#### Participants

Thirty preschool-aged children (*M*_*age*_ = 51.13 months, *SD* = 11.82, Range: 25–82 months; 13 girls) participated in the experiment. Effect sizes were gathered from previous studies on referent selection via MEB with children at 2 years of age, the youngest in this sample, which had consistently large effect sizes (*d*s > 1.0; Horst and Samuelson, 2008; Casler, 2014). To be conservative in determining a sample size, we used a smaller effect size in the large effect category: *d* = 0.90. A power analysis for a two-tailed *t*-test, with α = 0.05, revealed that we would need at least 21 participants to have 80% power to observe an effect. Thus, we decided to collect data for 3 months or until we reached 21 participants. These children had not participated in Experiment 1 or 2. Children were recruited through a lab preschool database at University of Wisconsin–Madison. An additional three children participated but were not included in the final sample due to fussiness. All children received a storybook as a thank-you for their participation. Informed consent was obtained from all participants.

#### Stimuli and Apparatus

This experiment used familiar and novel objects from Experiment 1. The experiment was presented on an iPad or laptop using a presentation application. Children’s selections were recorded by the experimenter on paper.

#### Design and Procedure

Children were presented an abbreviated version of the No Interference condition of Experiment 2.

##### Learning phase: referent selection learning trials

Children were presented with a learning phase which consisted of target novel objects that were only labeled once (Figure 5). There were four novel objects and four novel labels; each word and object was only presented on one learning trial. That is, children were presented four learning trials like the trials presented in the No Interference condition of Experiment 2.

##### Retention phase: 5-min delay

Same as Experiments 1 and 2.

##### Testing phase: word-referent mapping memory task

Same as Experiments 1 and 2, with the exception that there were only four testing trials, one for each word in the learning phase.

### Results and Discussion

Experiment 3 was designed to determine whether the cognitive load of learning many new words led to faster forgetting of words mapped via MEB. Thus, we examined whether children: (a) used MEB during the learning phase; and (b) retained words mapped via MEB.

#### Performance in the Learning Phase

Children’s performance during the learning phase was measured as the proportion of target novel objects selected across the four learning trials (chance performance was 0.50, as novel objects were presented with one familiar object each). As in Experiments 1 and 2, we defined learners’ use of MEB as the mapping of novel words to novel objects rather than to familiar objects during the learning phase. Results revealed that children mapped the labels to target novel objects on a majority of the trials (*M_*proportion of novel object choices*_* = 0.967, *SD* = 0.13). Children’s performance was significantly different from chance, *t*(29) = 20.15, *p* < 0.001, *d* = 3.68, suggesting that children were using MEB to map words to objects.

#### Performance in the Testing Phase

Children’s performance during the testing phase was measured as the proportion of target novel objects selected across all four test trials (chance performance was 0.33, as target novel objects were presented with one familiar object and one distractor novel object each). Results revealed that children mapped the labels to target novel objects on a majority of the trials (*M_*proportion of novel object choices*_* = 0.78, *SD* = 0.27). Children’s performance was significantly different from chance, *t*(29) = 8.85, *p* < 0.001, *d* = 1.62. We also examined children’s forgetting (i.e., difference scores) of labels between the learning and testing phase (*M* = 0.19, *SD* = 0.27); a paired samples *t*-test comparing the two time points revealed that children exhibited significant forgetting between word mapping and testing, *t*(20) = 3.92, *p* = 0.001, *d* = 0.71. This effect size in forgetting was similar to what was observed in Experiments 1 and 2 (medium-large to larger effect sizes). The results of three ANOVAs, with difference score as the outcome variable and condition (Experiment 3 vs. one of the conditions in Experiment 2) as a fixed factor and age as a covariate, revealed no main effects condition, *p*s > 0.05. That is, there were no significant differences between the difference score in this experiment and the three difference scores in Experiment 2 (Figure 9); children forgot MEB mapped words at equivalent rates across the experiments. Indeed, these results provide evidence against the hypothesis that the cognitive demands of the learning phase of Experiments 1 and 2 was the primary cause of rapid forgetting of words mapped via MEB. Implications of these findings are outlined in the section “General Discussion” below.

## General Discussion

The goal of these experiments was to determine whether MEB promotes retention of words to a greater degree than another strategy of fast-mapping (e.g., words mapped via random selection without use of MEB, or some other unspecified strategy). In Experiment 1, children and adults relied on MEB to map novel words to objects in the learning phase, but only when words and objects were completely novel (M1 trials). That is, when children were presented with a second label for a novel object, they did not exhibit use of MEB over use of any other strategy in particular. In the testing phase, children and adults did not have improved retention for labels that were mapped via MEB (M1 labels) than for words mapped via another strategy (M2 labels); rather, there was no significant difference in retention of the two types of labels after the 5-min delay. Indeed, children and adults experienced more rapid forgetting across time for labels mapped via MEB. In Experiment 2, we replicated the results of Experiment 1 and varied levels of interference from a second label to rule out the possibility that interference was driving forgetting. In Experiment 3, we reduced the cognitive demands of the task, but still observed forgetting of words mapped via MEB. Taken together, these results suggest that the use of MEB during word mapping provides no benefit for later retention of novel words, compared to other, unspecified strategies that children and adults might be using to map words to objects in moments of referential ambiguity.

This work provides several theoretical contributions to the literature on children’s word learning; we highlight a few here. First, this work demonstrates that MEB does not yield stronger retention for word mappings after a delay. Although there are a small number of studies with young children that have examined MEB after the moment of learning (e.g., Horst and Samuelson, 2008; Axelsson et al., 2018), most of these studies demonstrate no retention after a brief delay (i.e., chance performance) without additional memory supports. In contrast, this work demonstrates that, even in situations when children are indeed able to retain information at the delayed test, use of MEB does not promote long-term retention of words relative to other alternative, unspecified disambiguation strategies.

Second, this work begins to isolate the mechanisms underlying children’s retention of disambiguated words across time. In particular, Experiment 2 provides evidence against interference as being a key mechanism that drives the faster forgetting of words mapped via MEB. Although interference is thought to be a mechanism that disrupts encoding (Bjork, 2003), thus resulting in weaker memory for new information, these studies demonstrate that children’s forgetting of new words across time must be the result of some other cognitive mechanism at work during word learning. Moreover, Experiment 3 provides evidence against general cognitive demands of this experimental task as a potential mechanism underlying the forgetting of words mapped via MEB.

### Why Does MEB Not Result in a Long-Term Memory Advantage?

There are several possible mechanisms that may contribute to children’s forgetting of words mapped via MEB. First, children’s tendency to use MEB has previously been attributed to endogenous, novelty-seeking behaviors (e.g., Horst et al., 2011a; Mather and Plunkett, 2012; Dysart et al., 2016). If children’s use of MEB is driven by a novelty-seeking preference, then MEB would not facilitate strong encoding of words; rather, the process of encoding would be negatively affected by this constraint. Word learning via novelty seeking could be detrimental to encoding for a number of reasons. It could be that, although the interference of multiple novel labels did not hinder children’s retention, encoding could be negatively affected by competitors at the time of encoding. If children’s search for a novel object upon hearing a novel label engages encoding for the familiar objects as well as the novel object (i.e., visual search to determine which object is most novel), the competition may inhibit encoding of the target and promote encoding of irrelevant objects and features. Horst and Samuelson (2008) found that decreasing children’s attention to distractor objects by increasing the salience of the target object reduced competition and facilitated infants’ retention for new words; they concluded that the role of competition during referent selection was influential for long-term retention.

Alternatively, it is also a possibility that children’s more significant forgetting of words mapped via MEB is related to the amount of cognitive effort that children put (or rather, do not put) into initial word mapping. That is, if MEB is the result of quickly applying a rule (Markman, 1989), this short-cut may lead children to invest less cognitive effort into encoding, resulting in diminished long-term retention. For instance, Vlach et al. (2012) presented children with a word learning task in which encoding and retrieving new words was either easy (simultaneous presentation condition) or difficult (spaced presentation condition). At the immediate test, children had higher performance in the easy condition. However, at a 15-min delayed post-test, children in the easy condition rapidly forgot words, and children in the difficult condition had the strongest retention of words. Thus, more cognitive effort during word learning led to greater long-term performance. Indeed, these findings are reflective of a larger body of literature on human memory; desirable difficulties theory suggests that struggling to retrieve information during learning often leads to stronger performance and long-term retention (e.g., Bjork, 1994; Kornell et al., 2009; Pyc and Rawson, 2010; Bjork and Bjork, 2011; Vlach et al., 2012).

The current work also rules out potential explanations for previous research showing relations between children’s referent selection abilities and retention of new words. For instance, Bion et al. (2013) employed a looking-while-listening paradigm with infants at 18, 24, and 30 months of age in order to examine children’s disambiguation performance using MEB as well as their immediate retention of novel words. They observed a significant correlation between young children’s performance in disambiguation instances and their performance in retention trials at an immediate test. One possible explanation for these findings is that being quick to determine a referent (such as applying MEB) is what drives both referent selection and retention abilities across development. However, the current work, which investigated causal mechanisms of forgetting via MEB rather than mere correlational relations, suggests that being quick to select a referent could actually be detrimental to long-term word learning. Consequently, the relations observed in previous research (Bion et al., 2013) are likely to be due to mechanisms other than MEB, such as alternative word learning strategies or techniques.

Could children’s MEB use, under different circumstances, result in higher retention? This is an important question to pursue in future research. It may be that making the fast-mapping process slower, as well as creating an environment that engages children in more cognitive effort during encoding, can improve the degree to which MEB promotes long-term word learning. For instance, research has shown that providing children with more cues during fast mapping can improve the degree to which they remember words (Vlach and Sandhofer, 2012). This could be because the cues slow down encoding, require more cognitive effort to process during learning, and serve as retrieval supports across timescales.

In summary, the current studies highlight the need to bridge research on referent selection with children’s long-term memory for words, an area of work that has been under-studied for decades (Vlach, 2019). Much of this recent work, including the current experiments, has demonstrated that immediate performance in a word learning task does not always equate to performance over time (Vlach et al., 2012; Vlach and Sandhofer, 2014). Critically, the current work suggests that we should be careful to assume that the rules children might apply during referent selection are beneficial for long-term word learning. Indeed, these strategies may be quick tools for encoding, but could come at the cost of long-term learning.

## Data Availability Statement

The raw data supporting the conclusions of this article will be made available by the authors, without undue reservation.

## Ethics Statement

The studies involving human participants were reviewed and approved by the University of Wisconsin–Madison IRB. Written informed consent to participate in this study was provided by the participants’ legal guardian/next of kin.

## Author Contributions

CB and HV conceived of the research question and experimental design. CB directed the data collection and data cleaning, and took the lead role in conducting the data analysis, and writing the manuscript, with the assistance of HV. Both authors contributed to the article and approved the submitted version.

## Conflict of Interest

The authors declare that the research was conducted in the absence of any commercial or financial relationships that could be construed as a potential conflict of interest.

## Publisher’s Note

All claims expressed in this article are solely those of the authors and do not necessarily represent those of their affiliated organizations, or those of the publisher, the editors and the reviewers. Any product that may be evaluated in this article, or claim that may be made by its manufacturer, is not guaranteed or endorsed by the publisher.

## References

[B1] Au T. K. F. Glusman M. (1990). The principle of mutual exclusivity in word learning: to honor or not to honor? *Child Dev.* 61 1474–1490. 10.2307/1130757 2245739

[B2] Axelsson E. L. Swinton J. Winiger A. I. Horst J. S. (2018). Napping and toddlers’ memory for fast-mapped words. *First Lang.* 38 582–595. 10.1177/0142723718785490

[B3] Bion R. A. Borovsky A. Fernald A. (2013). Fast mapping, slow learning: disambiguation of novel word–object mappings in relation to vocabulary learning at 18, 24, and 30 months. *Cognition* 126 39–53. 10.1016/j.cognition.2012.08.008 23063233 PMC6590692

[B4] Bjork E. L. Bjork R. A. (2011). “Making things hard on yourself, but in a good way: creating desirable difficulties to enhance learning,” in *Psychology and the Real World: Essays Illustrating Fundamental Contributions to Society*, eds Gernsbacher M. A. Pew R. W. Hough L. M. Pomerantz J. R. (New York, NY: Worth Publishers), 56–64.

[B5] Bjork R. A. (1994). “Memory and metamemory considerations in the training of human beings,” in *Metacognition: Knowing About Knowing*, eds Metcalf J. Shimura A. P. (Cambridge, MA: MIT Press), 185–205.

[B6] Bjork R. A. (2003). “Interference and forgetting,” in *Encyclopedia of Learning and Memory*, 2nd Edn, ed. Byrne J. H. (New York, NY: Macmillan Reference USA), 268–273.

[B7] Carey S. Bartlett E. (1978). *Acquiring a Single New Word*, Vol. 15. Papers and Reports on Child Language Development (Department of Linguistics, Stanford University). Stanford, CA, 17–29.

[B8] Casler K. (2014). New tool, new function? Toddlers’ use of mutual exclusivity when mapping information to objects. *Infancy* 19 162–178. 10.1111/infa.12044

[B9] Dysart E. L. Mather E. Riggs K. J. (2016). Young children’s referent selection is guided by novelty for both words and actions. *J. Exp. Child Psychol.* 146 231–237. 10.1016/j.jecp.2016.01.003 26897305

[B10] Evey J. A. Merriman W. E. (1998). The prevalence and the weakness of an early name mapping preference. *J. Child Lang.* 25 121–147. 9604571 10.1017/s030500099700336x

[B11] Fenson L. Dale P. S. Reznick J. S. Bates E. Thal D. J. Pethick S. J. (1994). Variability in early communicative development. *Monogr. Soc. Res. Child Dev.* 59 1–173. 7845413

[B12] Golinkoff R. M. Mervis C. B. Hirsh-Pasek K. (1994). Early object labels: the case for a developmental lexical principles framework. *J. Child Lang.* 21 125–155. 10.1017/S0305000900008692 8006089

[B13] Gurteen P. M. Horne P. J. Erjavec M. (2011). Rapid word learning in 13-and 17-month-olds in a naturalistic two-word procedure: looking versus reaching measures. *J. Exp. Child Psychol.* 109 201–217. 10.1016/j.jecp.2010.12.001 21216414

[B14] Halberda J. (2003). The development of a word-learning strategy. *Cognition* 87 B23–B34. 10.1016/S0010-0277(02)00186-5 12499109

[B15] Horst J. S. Samuelson L. K. (2008). Fast mapping but poor retention by 24-month-old infants. *Infancy* 13 128–157. 10.1080/15250000701795598 33412722

[B16] Horst J. S. Samuelson L. K. Kucker S. C. McMurray B. (2011a). What’s new? Children prefer novelty in referent selection. *Cognition* 118 234–244. 10.1016/j.cognition.2010.10.015 21092945 PMC3022084

[B17] Horst J. S. Scott E. J. Pollard J. A. (2011b). The role of competition in word learning via referent selection. *Dev. Sci.* 13 706–713. 10.1111/j.1467-7687.2009.00926.x 20712736

[B18] Kornell N. Hays M. J. Bjork R. A. (2009). Unsuccessful retrieval attempts enhance subsequent learning. *J. Exp. Psychol.: Learn. Mem. Cogn.* 35:989. 10.1037/a0015729 19586265

[B19] Kucker S. C. (2013). *The Role of Vocabulary Knowledge and Novelty Biases in Word Learning: Exploring Referent Selection and Retention in 18-to 24-Month-Old Children and Associative Models.* Ph.D. thesis, University of Iowa, IA, Iowa. 10.17077/etd.ahssj6dt

[B20] Markman E. M. (1989). *Categorization and Naming in Children: Problems of Induction.* Cambridge, MA: Bradford/MIT Press.

[B21] Markman E. M. (1990). Constraints children place on word meanings. *Cogn. Sci.* 14 57–77. 10.1207/s15516709cog1401_4

[B22] Markman E. M. Wachtel G. F. (1988). Children’s use of mutual exclusivity to constrain the meanings of words. *Cogn. Psychol.* 20 121–157. 10.1016/0010-0285(88)90017-5 3365937

[B23] Mather E. Plunkett K. (2009). Learning words over time: the role of stimulus repetition in mutual exclusivity. *Infancy* 14 60–76. 10.1080/15250000802569702 32693468

[B24] Mather E. Plunkett K. (2012). The role of novelty in early word learning. *Cogn. Sci.* 36 1157–1177. 10.1111/j.1551-6709.2012.01239.x 22436081

[B25] McMurray B. Horst J. S. Samuelson L. K. (2012). Word learning emerges from the interaction of online referent selection and slow associative learning. *Psychol. Rev.* 119 831–877. 10.1037/a0029872 23088341 PMC3632668

[B26] Merriman W. E. Bowman L. L. MacWhinney B. (1989). The mutual exclusivity bias in children’s word learning. *Monogr. Soc. Res. Child Dev.* 54 1–132. 10.2307/1166130 2608077

[B27] Merriman W. E. Marazita J. Jarvis L. H. (1993). Four-year-olds’ disambiguation of action and object word reference. *J. Exp. Child Psychol.* 56 412–430. 10.1006/jecp.1993.1042 8301246

[B28] Mervis C. B. Bertrand J. (1994). Acquisition of the novel name–nameless category (N3C) principle. *Child Dev.* 65 1646–1662. 10.1111/j.1467-8624.1994.tb00840.x 7859547

[B29] Mervis C. B. Golinkoff R. M. Bertrand J. (1994). Two-year-olds readily learn multiple labels for the same basic-level category. *Child Dev.* 65 1163–1177. 10.1111/j.1467-8624.1994.tb00810.x 7956472

[B30] O’Connor R. J. Riggs K. J. (2019). Adult fast-mapping memory research is based on a misinterpretation of developmental-word-learning data. *Curr. Direct. Psychol. Sci.* 28 528–533. 10.1177/0963721419858426

[B31] Pyc M. A. Rawson K. A. (2010). Why testing improves memory: mediator effectiveness hypothesis. *Science* 330 335–335. 10.1126/science.1191465 20947756

[B32] Soderstrom N. C. Bjork R. A. (2015). Learning versus performance: an integrative review. *Perspect. Psychol. Sci.* 10 176–199. 10.1177/1745691615569000 25910388

[B33] Spiegel C. Halberda J. (2011). Rapid fast-mapping abilities in 2-year-olds. *J. Exp. Child Psychol.* 109 132–140. 10.1016/j.jecp.2010.10.013 21145067

[B34] Vlach H. Sandhofer C. M. (2012). Fast mapping across time: memory processes support children’s retention of learned words. *Front. Psychol.* 3:46. 10.3389/fpsyg.2012.00046 22375132 PMC3286766

[B35] Vlach H. A. (2019). Learning to remember words: memory constraints as double-edged sword mechanisms of language development. *Child Dev. Perspect.* 13 159–165. 10.1111/cdep.12337

[B36] Vlach H. A. Ankowski A. A. Sandhofer C. M. (2012). At the same time or apart in time? The role of presentation timing and retrieval dynamics in generalization. *J. Exp. Psychol. Learn. Mem. Cogn.* 38 246–254. 10.1037/a0025260 21895392 PMC3302959

[B37] Vlach H. A. Sandhofer C. M. (2014). Retrieval dynamics and retention in cross-situational statistical word learning. *Cogn. Sci.* 38 757–774. 10.1111/cogs.12092 24117698 PMC3979515

[B38] Woodward A. L. (2000). “Constraining the problem space in early word learning,” in *Becoming a Word Learner: A Debate on Lexical Acquisition*, eds Golinkoff R. Hirsh-Pasek K. Bloom L. Hollich G. Smith L. Woodward A. L. (Oxford: Oxford University Press), 81–114.

